# The MiLES intervention targeting employers to promote successful return to work of employees with cancer: design of a pilot randomised controlled trial

**DOI:** 10.1186/s13063-020-04288-0

**Published:** 2020-04-28

**Authors:** Michiel A. Greidanus, Angela G. E. M. de Boer, Angelique E. de Rijk, Monique H. W. Frings-Dresen, Sietske J. Tamminga

**Affiliations:** 1 Amsterdam UMC, University of Amsterdam, Coronel Institute of Occupational Health, Amsterdam Public Health research institute, Meibergdreef 9, Amsterdam, The Netherlands; 2 Department of Social Medicine, Faculty of Health, Medicine and Life Sciences, Research Institute Primary Care and Public Health (CAPHRI), Maastricht University, Duboisdomein 30, Maastricht, The Netherlands

**Keywords:** Work, Cancer, Employee, Employer, Manager, Cancer survivors, Clinical trial protocol, Return to work, Pilot randomised controlled trial protocol, Intervention

## Abstract

**Background:**

Employers express a need for support to facilitate the return to work (RTW) process of employees with cancer. We have developed the MiLES intervention, an online toolbox targeting employers during the RTW of employees with cancer. To evaluate the MiLES intervention, we propose the design of a pilot randomised controlled trial (RCT). The aim of this pilot is to determine whether a future RCT to study the effectiveness of this intervention on successful RTW of employees with cancer is feasible. Secondary aims are to obtain preliminary results on the effectiveness of the intervention and to determine the sample size needed in a future definitive RCT.

**Methods:**

A pilot RCT with a 6-month follow-up will be conducted. Using medical specialists at Dutch hospitals, we aim to enrol 90 participants diagnosed with cancer (<2 years earlier) aged 18–63 years who are in paid employment with an employer and who are currently sick-listed or partly sick-listed for <1 year. Participants randomised to the intervention group will be asked to inform their employer about the online toolbox supporting employers during the RTW process of employees with cancer. Participants in the control group will receive ‘care as usual’ from their employer. All measures will be assessed at the level of the employee using questionnaires at baseline and after 3 and 6 months of follow-up. The feasibility of a future RCT will be determined using criteria concerning method-related uncertainties and acceptability of the study protocol. The primary effect measure will be successful RTW (that is, RTW perceived as being successful by the cancer survivor themselves). This effect measure will be used to perform the sample size calculation for a future definitive RCT.

**Discussion:**

The design is proposed to determine the feasibility to study the effectiveness of the MiLES intervention targeting employers on the successful RTW of employees diagnosed with cancer. This pilot RCT can increase the probability of a successful future definitive RCT on the effectiveness of the intervention and potentially obviate the need to carry out an unfeasible and resource-intensive study.

**Trial registration:**

Dutch Trial Register (NTR): NL6758, NTR7627. Registered on 30 October 2018.

## Background

Of the 14.1 million persons who are diagnosed with cancer each year, approximately 50% are of working age [1, 2]. With improved survival rates taken into account, the consequences of a diagnosis of cancer for a person’s work participation are becoming increasingly important [3]. A relatively high percentage (8–40%) of employed cancer survivors are not able to stay in or return to work (RTW) [4]. Personal, health- and work-related factors may hamper the work participation of cancer survivors [5–8]. Work-related factors include, for example, the negative attitudes of employers and co-workers, heavy job demands, stigma and discrimination [5, 7, 8]. Being unable to work is unfortunate for cancer survivors as work provides social interaction and financial security and is also associated with a higher quality of life [9, 10]. In addition, at both the organisational and the societal level, improving the work participation of cancer survivors might reduce the economic burden of a cancer diagnosis [11]. Increasing the work participation of cancer survivors is therefore a relevant topic both for the individual and for organisations and society at large [9–11].

A number of work-related interventions have been developed to facilitate the work participation of cancer survivors—for example, occupational training, vocational counselling, tailored work-related support from the clinic, or work accommodations [12–15]. These interventions are mainly focussed on amending the cancer survivor’s behaviour and drawing attention to work-related matters in clinical care; however, the results so far are at best inconclusive [12, 14]. Future work-related interventions should therefore also include other stakeholders in the RTW process [14, 16]. Employers are repeatedly acknowledged as one of the main stakeholders in the RTW process [17–21], but they find that supporting the RTW of an employee with cancer is both complex and demanding [22]. Many different aspects potentially prevent employers from providing appropriate support—for example, national and organisation policies, employers’ lack of knowledge about cancer, and the conflicting interests of the employee with cancer versus that of the organisation [16].

We used the intervention mapping approach to develop the MiLES intervention, to support employers during the RTW of employees with cancer ('MiLES is an abbreviation for 'the Missing Link: optimising return to work of Employees diagnosed with cancer, by Supporting employers') [23, 24]. This web-based intervention, which is an ‘online toolbox’, aims to amend the behaviour of the employer during the various RTW phases of an employee with cancer: 1) the period between disclosure of the employee’s illness to the employer and the first treatment; 2) the period of sick leave during the employee’s treatment; 3) the period in which the concrete planning and preparation of the employee’s RTW take place; and 4) the period after RTW [24]. Since employees with cancer may experience their work disability differently, which may result in different support needs from their employer, the online toolbox allows for tailoring to this experience as three ‘types of experiences’ are addressed: 1) an emotional employee with cancer, in which intense emotions such as sadness and anger can alternate quickly; 2) the employee with cancer who wants little attention for their health situation, and prefers to be involved in work for as long as possible, and return to work as quickly as possible; and 3) the employee with cancer who starts looking differently at work and life, and gives other priorities due to their illness [24, 25]. To the best of our knowledge, this will be the first scientific study on an intervention solely targeting employers during the RTW process of an employee diagnosed with cancer [26].

The primary aim of the online toolbox is to promote the successful RTW of employees diagnosed with cancer [24]. As ‘just’ returning to work does not guarantee that an employee is satisfied with their RTW [27, 28], a newly developed questionnaire will be used to quantify the successfulness of RTW: the Successful Return To Work questionnaire for Cancer Survivors (I-RTW_CS). This questionnaire incorporates elements that are perceived to be most important for successful RTW according to employees with cancer. Since the MiLES intervention will intervene at the level of the employer and the outcomes will be measured at the level of the employee with cancer, an innovative study design is needed. For example, due to the General Data Protection Regulation, the exchange of health-related information between employee and employer is not allowed, which makes it difficult to develop a study design that is easily accessible and compliant with those regulations.

Due to the innovative character of the online toolbox, the outcome measure and the study design, a pilot study prior to a randomised controlled trial (RCT) to study the effectiveness of the online toolbox is highly recommended [29, 30]. This will enable protocol and method-related uncertainties about such an RCT to be studied, which in turn can increase the probability of a successful RCT and potentially obviate the carrying out of an unfeasible and resource-intensive study [30–32]. Other future studies on RTW interventions involving an employee’s employer might also benefit from this study design.

The objective of this paper is to describe the design of a pilot RCT to evaluate the MiLES intervention targeting employers during the RTW of employees with cancer. The aim of this pilot is to determine whether an RCT to study effectiveness of this intervention is feasible in terms of recruitment, reach and acceptability of the study protocol. Secondary objectives are to obtain preliminary results on the effectiveness of the intervention on successful RTW and to determine the sample size needed in a future definitive RCT to study the effectiveness of the intervention.

## Methods

**Fig. 1:**
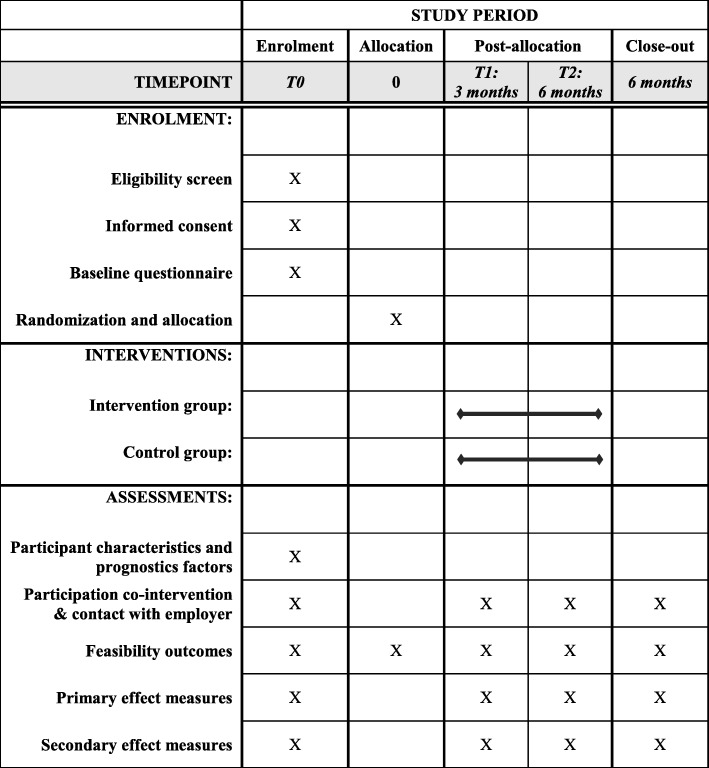
A schedule of enrolment, intervention and assessments

**Fig. 2:**
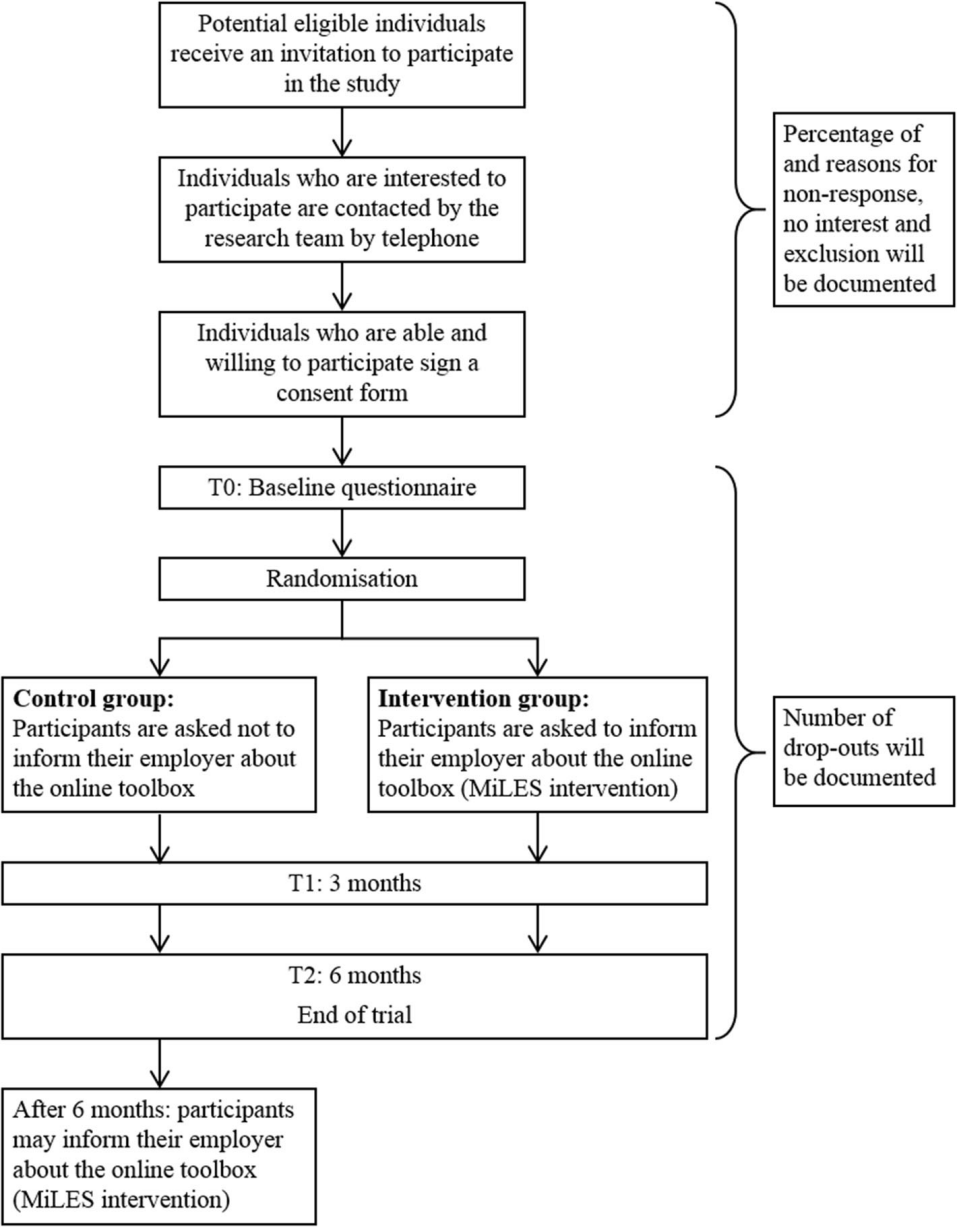
A participant flowchart of the pilot randomised controlled trial

The study will be conducted as a non-blinded pilot RCT with a follow-up of 6 months. A schedule of enrolment, intervention and the timing of measurements is shown in Fig. 1. The participant flowchart is shown in Fig. 2. The study will include employees diagnosed with cancer and will compare the MiLES intervention targeting the employer of included employees with a waiting-list control group in which the employer will not receive the intervention. The CONSORT 2010 statement for randomised pilot trials and the SPIRIT checklist were used to structure the design of this pilot-RCT [32–34]. The completed SPIRIT checklists can be found in Additional file 1.

### Participants

We aim to include 90 individuals. This number is thought to be sufficient to provide the appropriate information required to determine the feasibility of the study protocol and to calculate the targeted sample size for a definitive RCT to study the effectiveness of the online toolbox on successful RTW. Both are stated as main considerations for determining the sample size of pilot trials [29, 31]. The inclusion criteria will be:

- Of working age (between 18 and 63 years);
- Diagnosed with cancer <2 years earlier;
- In paid employment under a temporary (>6 months remaining) or permanent contract on a part-time, full-time or flexible basis;
- Currently sick-listed or partially sick-listed for <1 year;
- Able to complete 3 questionnaires in the following 6 months, as assessed by their medical specialist on the basis of their current health and expectations about their health in the future;
- Able to understand, speak and read Dutch sufficiently;
- Having already informed the employer about the diagnosis of cancer. The aim of this criterion is to avoid putting unintended pressure on the participant to disclose the cancer diagnosis, as the online toolbox provides the participant’s employer with general information about how to support employees with cancer.

No restrictions will be applied for any co-interventions of the participant and/or the participant’s employer.

Participants will be recruited via medical specialists at various hospitals in the Netherlands, starting in March 2019. Medical specialists will send an invitation letter, information sheet, approach form and return envelope to potential eligible individuals, based on their age, diagnosis, time since diagnosis, their current health and expectations about their health in the future (to prevent patients being invited with the unethical request to participate in a study primarily aimed at enhancing successful RTW while their prognosis would most likely not allow them to work in the future). These materials, and all other materials that will be used in the study, are written by a professional text writer in collaboration with medical specialists and the authors, who have comprehensive experience with text writing and questionnaire development for employees with cancer and employers. All individuals will be asked to indicate on the approach form whether they meet the inclusion criteria and whether they are interested in participating in the study and, if not, to indicate why they are not interested. Individuals will be asked to return this form to the first researcher of the study (MAG). If an individual is interested, they will be contacted by the research team to explain the study, to check the individual’s eligibility based on all the inclusion criteria and to give the individual the opportunity to ask questions. If an individual is eligible for the study and wants to participate, a digital informed consent form will be sent to them. After signing the form, the participant will be assigned a unique number in chronological order of inclusion, starting at 001, to guarantee the blindness of participant’s data. After inclusion, participants will be asked to complete the baseline questionnaire and will thereafter be randomised to the intervention or the control group.

### Randomisation

Participants will be randomised with variable block randomisation to the control or the intervention group using the electronic data capture system CASTOR [35]. The allocation of participants will be definitive, and the researchers will not be able to influence the process. The randomisation rate for the intervention and control group will be set at 2:1 in order to have a larger sample size in the intervention group and thus provide more information about the criteria for the feasibility of the study design. To prevent unequal distribution over the groups, randomisation will be controlled for the participant’s RTW status, namely having performed work activities in their own work or in replacement work during the previous 4 weeks (yes versus no). This is important since it is not possible to evaluate the effect of the intervention on successful RTW when RTW itself (being a prerequisite for successful RTW) is already unequally distributed over the two groups after randomisation. Neither the participant nor the researchers will be blinded for the randomisation. We thought it would be unethical to not inform eligible employees with cancer about the intervention before their decision to participate in the study.

#### The MiLES intervention targeting the participant’s employer

The MiLES intervention will consist of an online toolbox targeting the participant’s employer [24]. ‘Employer’ refers to the person who is, as direct supervisor, human resource manager or case manager, in direct contact with the participant and thus the person who can provide RTW support. In the Netherlands, where this study will take place, the employer is responsible—together with the employee and an occupational physician in a consultative role—for sickness absence guidance and RTW guidance for a period of 2 years [36]. To improve readability, ‘employer’ is used throughout this paper.

The online toolbox is an open-access website containing information, videos and conversation checklists to support employers during the RTW process of an employee diagnosed with cancer. The content of the online toolbox is tailored per RTW phase and per ‘type of experience’ of the employee with cancer [24, 25]. The online toolbox is accessible via a URL that will be ‘hidden’ throughout the study period (i.e. the URL will not be traceable via Google or any other online search engine). A comprehensive overview of the development, aim and various components of the toolbox has been published elsewhere [24]. In short, the toolbox has been developed with input from various scientific sources and stakeholders: interviews with 30 employers [22], a systematic review of international literature [16], a Delphi study with 29 employees with cancer and 23 employers [37], interviews with eight eHealth experts, focus groups with seven employees with cancer and seven occupational physicians specialised in cancer, and walk-through interviews with five employers.

#### Intervention group

Participants randomised to the intervention group will be asked to inform their employer about the online toolbox either by letter or by email. Participants who prefer to inform their employer by email will receive a standard email from the first researcher of the study. This email will contain a secured PDF file with the following content: information about the participant’s involvement in the study; the URL of the online toolbox; and a promotional video (docu-fiction) to encourage the employer to visit the online toolbox. The PDF will be secured with a password because the PDF will refer to employees diagnosed with cancer. The password will be sent by participants to their employer via a text message. Participants who prefer to inform their employer by letter will be able to do so by post or personally. The standard letter will contain the same information as the abovementioned email, supplemented with QR codes and URLs of the online toolbox and the promotional video. In all cases, employers will be asked to watch the promotional video and use the online toolbox throughout the RTW process of their employee with cancer (i.e. the participant).

#### Control group

Participants randomised to the control group will not be able to inform their employer about the online toolbox until a period of 6 months has elapsed. Participants will therefore receive ‘care as usual’ from their employer. The trial and data collection will end at 6 months. Thereafter, participants in the control group will be enabled to inform their employer about the online toolbox. This will therefore not contaminate the comparison between the intervention and control group. When a participant in the control group wants to inform their employer after 6 months, they will be able to use the same materials as the participants randomised to the intervention group.

### Data collection

Study parameters will be assessed using questionnaires completed either electronically (via the electronic data capture system CASTOR [35]) or on paper. Questionnaires will be sent to participants at baseline (before randomisation; T0) after 3 months of follow-up (T1) and after 6 months of follow-up (T2). If a participant fails to complete one of the questionnaires, they will be reminded by email and telephone after 1 and 2 weeks, respectively.

#### Participant characteristics

At T0, the questionnaire will include questions about the participant’s characteristics and work (for example age, gender, diagnosis, time since diagnosis, level of education, treatment, treatment duration, work status, type of contract, type of work, sector and size of the organisation in which the participant works), and a question to determine the participant’s type of experience with cancer, based on Tiedtke and colleagues [25]. All questionnaires will also assess the participant’s involvement in any co-intervention and the number of contact moments with the employer since diagnosis (at T0) or in the previous 3 months (at T1 and T2) (open questions). Finally, the RTW phase of participants will be asked in all questionnaires [24].

#### Feasibility of an RCT

To determine the feasibility of studying the effectiveness of the online toolbox using an RCT, the following protocol and method-related measures will be tracked: reach (number of individuals who received an invitation to participate in the study), recruitment rate (percentage of potential eligible individuals who are included) and retention rate (percentage of participants not lost before follow-up) [29, 38]. In addition, the appropriateness of the inclusion criteria and procedures for randomisation and informing the employer will be measured by recording the number of individuals who were not interested in participating in the study or who dropped out during the different phases of the study, and their reasons for non-participation or dropping out. Finally, we will ask participants in the intervention group at 6 months of follow-up whether they have actually informed their employer about the online toolbox. As such, we will determine whether or not participants are willing to inform their employer about the online toolbox to determine the acceptability of the study protocol [38]. Knowing whether or not participants actually informed their employer about the online toolbox is also important since a prerequisite for employers being able to use the online toolbox is that they are informed by the employee in the first place.

#### Effect evaluation

Measures for effect evaluation will be assessed at each time point (i.e. T0, T1 and T2). The primary effect measure will be the combined outcome measure ‘successful RTW’. This effect measure is a combination of two components:

1. RTW, about which all participants will be asked. RTW is defined as ‘having performed work activities in their own work or in adapted work during the previous 4 weeks’. We will ask participants the following: ‘Have you performed any work activities in the past 4 weeks? This can be either your own original work activities, or adapted work activities replacing your original work [yes/no].’
2. *I-RTW_CS*, which only those participants who returned to work will be asked. This measure will be a questionnaire on the successfulness and importance of seven items that constitute successful RTW. Each item’s success score (on a 6-point rating scale) will be weighted by its perceived importance (on a 5-point rating scale). For this, the success score will be multiplied by the importance score, resulting in the item’s weighted score. All weighted scores will be summed and divided by the sum of all items’ importance scores, resulting in the *I- RTW_CS* score.

Secondary effect measures will be:

1. The Quality of Working Life Questionnaire for Cancer Survivors (QWLQ-CS) [39]. This questionnaire will only be completed by participants who returned to work according to the abovementioned definition of RTW, since the questionnaire is about the experiences and perceptions of cancer survivors in the work environment in the previous 4 weeks [39]. The answer option ‘does not apply’ for employer-related questions will be omitted for this study since this option is intended for self-employed cancer survivors, who will be excluded for participation in the current study. The QWLQ-CS has been tested among employees with cancer and reveals good psychometric properties among employed cancer survivors [40, 41].
2. Unwanted work changes since diagnosis (at T0) or in the previous 3 months (at T1 and T2).

### Statistical analysis

Data will be analysed using SPSS 24.0 (IBM, NY, USA). All data will be analysed according to the intention-to-treat principle. All participants' characteristics, feasibility data, and primary and secondary effect measures for effect evaluation will be assessed and presented using descriptive statistics. *P* values ≤0.05 will be considered statistically significant.

#### Feasibility of RCT

There are no guidelines in the international literature for assessing the feasibility of an RCT [38, 42]. The present research team formulated the main uncertainties concerning the feasibility of a definitive RCT to study the effectiveness of the online toolbox and specified criteria for each of these uncertainties: ≥70% of the individuals who gave permission for telephone contact are willing to participate in the study (criteria appropriateness inclusion criteria, criteria recruitment), ≤20% do not want to participate due to the randomisation procedure or are lost to follow-up due to randomisation in the control group (criteria appropriateness protocol), ≤20% of the participants are not willing to inform their employer about the online toolbox after randomisation into the intervention group (criteria appropriateness protocol), ≤20% of the participants are lost to follow-up (criteria retention rate), and the sample size number of 90 participants are included within the recruitment period of 6 months, starting when the first individual is invited by their treating physician (criteria reach). An RCT to study the effectiveness of the online toolbox will be considered feasible when all of the criteria are met. If not, adjustments for the study protocol will be formulated.

#### Effect evaluation

Equal distribution of the randomisation at baseline will be examined for differences between the intervention and the control group using Student’s *t* tests for continuous variables and Chi-square tests for categorical variables. In the case of a statistically significant unequal distribution, it will be determined whether this variable is a prognostic factor for the primary effect measure. This will be determined for both components of the primary effect measure separately; for RTW on the basis of two reviews [43, 44], and for I-RTW_CS on the basis of a study that is currently being developed (see below for how we will correct for unequal distribution). The statistical power of this pilot study will only allow for the correction of a maximum of one baseline characteristic per component of the primary effect measure [45]. In the case of no statistically significant unequal distribution at baseline, the relative risk (RR) will be determined for RTW at T1 and T2. A longitudinal multilevel analysis will be performed with the group classification as an independent variable and I-RTW_CS scores at the different time points (T0, T1 and T2) as dependent variables. It is hypothesised that: 1) participants in the intervention group will return to work more often than participants in the control group; and 2) that the degree of successfulness of RTW (I-RTW_CS score) of the subgroup of participants that did return to work will be significantly higher in the intervention group than in the control group.

In the case of a statistically significant unequal distribution of a prognostic factor for RTW or I-RTW_CS, this prognostic factor will be used as a covariate for the analysis of that effect measure. A logistic regression analysis will be used to examine the differences between the intervention and the control group with regard to RTW. For the subgroup of participants that did return to work, a longitudinal multilevel analysis will be performed to examine the differences between both groups in I-RTW_CS scores. The outcomes of both adjusted and unadjusted analyses will be presented.

For the secondary effect measures, a longitudinal multilevel analysis will be performed with the group classification as an independent variable and QWLQ-CS at the different time points (T0, T1 and T2) as dependent variables. The RR will be determined for unwanted work changes (i.e. at least one unwanted work change) at T0, T1 and T2.

#### Sample size calculation for the RCT

For both components of the primary effect measure a separate sample size calculation will be made using nQuery Advisor® 7.0. The first sample size calculation will be based on the RTW percentages of the intervention and the control group. The second sample size will be based on the average and variance of the intervention and the control group concerning I-RTW_CS scores. Here, RTW rates will also be taken into account since the I-RTW_CS will only be measured in a subgroup of participants who did return to work. For both sample size calculations, the following additional data will be used: percentage of participants lost to follow-up in this pilot study, 80% power, a *P* value of < 0.05, and a randomisation ratio for intervention versus control group of 1:1. Once the sample size for both components has been calculated, the final sample size will be the highest of both.

## Discussion

The primary aim of this pilot RCT is determine whether an RCT to study effectiveness of the MiLES intervention is feasible in terms of recruitment, reach and acceptability of the study protocol. The design of this pilot RCT is described in this paper, including criteria for feasibility. We hypothesise that an RCT to study the effectiveness of this intervention is feasible with the current design. Secondary aims of the pilot are to obtain preliminary results on the effectiveness of the intervention on successful RTW and to determine the sample size needed in a future definitive RCT to study the effectiveness of the intervention. For this, we hypothesise that employees in the intervention group will return to work more often than employees in the control group, and that their degree of successful RTW will be higher.

### Methodological considerations

The main strength of the proposed design is its innovativeness; it will enable the measuring of outcomes in a population (i.e. employees diagnosed with cancer) that differs from the population whose behaviour is intended to be changed through the intervention (i.e. employers). Privacy regulations concerning the exchange of health-related information and ethical concerns will be fully respected by not collecting data at the level of the employer and by excluding employees who have not yet informed their employer about their diagnosis of cancer. Employees in the intervention group will be asked to inform their employer about the online toolbox. As such, the effectiveness of the online toolbox, which aims to amend the behaviour of the employer, will be measured at the level of the employee.

A drawback of not collecting employer data in the study is that we will lack exposure data of the online toolbox and will not be able to compute ‘dose–response’ relationships. Alternative methods to determine exposure data were deemed unsuitable or unreliable—that is, web analytics services would require employers to log on to the online toolbox, which may increase the threshold for employers to use the online toolbox. Besides, asking employees to estimate their employer’s use of the online toolbox would be unethical and assumedly unreliable. Instead, priority has been given to stimulate employers to use the online toolbox by making procedures for informing the employer about the online toolbox approachable (materials are written by a professional text writer, and the online toolbox is an open website without the need to log in) and tempting (by sending a short docu-fiction). Stimulating employers to use the online toolbox is thought to be important since it turned out to be challenging to involve employers in a previous RTW intervention [46]. With the procedures proposed in the current design, the involvement of employers is thought to be facilitated to optimise the exposure of the online toolbox to the employer. However, next to this pilot RCT with employees with cancer, a future study explicitly focusing on the employers’ use of and perspectives on the online toolbox is recommended. In addition, given that cancer treatment may last for months, which may lead to considerable and lengthy fluctuations in successful RTW [14], we recommend a future study on the effectiveness of the online toolbox to adopt a follow-up period of at least 1 year.

Employees randomised to the control group will not inform their employer about the online toolbox and will therefore receive care as usual from their employer. It might be that care as usual is still fairly good care in the current design, since study participation might lead to changed behaviour towards their employer (for example, asking more questions or demanding more support), and since the Dutch Gatekeeper Improvement Act stimulates active employer involvement regarding the RTW process [47]. However, the Dutch legislation is very general and of limited effect [47], which endorses the added value of the online toolbox for the intervention group. Employers and employees diagnosed with cancer have expressed specific and strong need for more extensive guidance for employers [18, 22]. This is especially the case for relatively small organisations, which tend to have less access to supportive resources (e.g. occupational physicians and human resource services) [37, 48, 49] and to current RTW programmes [49, 50], and struggle with their far-reaching financial and other responsibilities under the Dutch legal system [47]. Including employees and employers of small organisations in interventions is therefore of great importance [49, 50]; this is also the case for the current pilot RCT.

To assess the feasibility of a definitive RCT to study the effectiveness of the online toolbox, the main uncertainties for feasibility were discussed by the researchers and formulated as criteria for feasibility. This procedure for evaluating the feasibility of a definitive RCT is acknowledged in international research [38, 42]. However, there is serious inconsistency concerning the design, execution and evaluation of pilot studies, which are even interchangeably termed as exploratory or feasibility trails [38]. More specific guidelines concerning the design, execution, analysis and reporting of pilot studies are therefore needed, and might be published shortly [51, 52].

### Impact of results

This pilot RCT can increase the probability of a successful future definitive RCT on the effectiveness of the intervention and potentially obviate the carrying out of an unfeasible and resource-intensive study [30–32]. Other future RTW interventions might also benefit from the outcomes of the proposed pilot RCT. First, several method-related measures will provide insight into the feasibility of studying the effect of RTW interventions targeting employers on the work-related outcomes of employees on sick leave. The present study will, for example, provide knowledge on employees’ willingness to inform their employer about a supportive intervention aiming to amend the behaviour of the latter. Second, the study will provide insight into the usability of the innovative combined outcome measure of successful RTW. This is important, since the effectiveness of work-related interventions is currently mostly evaluated on the basis of time until full or part RTW [14, 53], even though this measure does not necessarily correspond to employees’ views of successful RTW [27]. It is argued that work-related issues and evaluation should be approached in a patient-centred manner, taking into account individual work-related goals [28]. The effect measure to be used in the present study includes several items that constitute successful RTW, and weights the items on the basis of their relative importance for the individual employee. Knowledge on the usability of and insight into the psychometric properties of this patient-centred weighted outcome measure of successful RTW is an important step towards a more meaningful evaluation of RTW interventions.

## Trial status

The study was registered in the Dutch Trial Register on 30 November 2018 (NL6758, NTR7627) at www.www.trialregister.nl/trial/6758*.* The current manuscript describes the same protocol as registered in the Dutch Trial Register (version 1.0, 30 November 2018). Recruitment of participants started in March 2019 and is expected to be completed in September 2019. The results of the pilot RCT are expected in 2020.

## Supplementary information

**Additional file 1.** Completed SPIRIT checklist.

## Data Availability

Data sharing is not applicable to this article as no datasets were generated or analysed during the current study. All data generated or analysed during the study will be included in the published results.

## Abbreviations

I-RTW_CS: Successful Return To Work Questionnaire for Cancer Survivors
MiLES: The missing link: optimising return to work of employees diagnosed with cancer, by supporting employers
QWLQ-CS: Cancer-specific Quality of Working Life Questionnaire
RCT: Randomised controlled trial
RR: Relative risk
RTW: Return to work

## References

[1] Ferlay J, Soerjomataram I, Dikshit R, Eser S, Mathers C, Rebelo M (2015). Cancer incidence and mortality worldwide: sources, methods and major patterns in GLOBOCAN 2012. Int J Cancer.

[2] Ferlay J, Steliarova-Foucher E, Lortet-Tieulent J, Rosso S, Coebergh JW, Comber H (2013). Cancer incidence and mortality patterns in Europe: estimates for 40 countries in 2012. Eur J Cancer.

[3] Duijts S, Dalton SO, Lundh MH, Horsboel TA, Johansen C (2017). Cancer survivors and return to work: current knowledge and future research. Psychooncology.

[4] Paltrinieri S, Fugazzaro S, Bertozzi L, Bassi MC, Pellegrini M, Vicentini M, et al. Return to work in European cancer survivors: a systematic review. Support Care Cancer. 2018;26(9):2983-94. 10.1007/s00520-018-4270-6 29845421

[5] Amir Z, Wynn P, Chan F, Strauser D, Whitaker S, Luker K (2010). Return to work after cancer in the UK: attitudes and experiences of line managers. J Occup Rehabil.

[6] Duijts SF, van Egmond MP, Spelten E, van Muijen P, Anema JR, van der Beek AJ (2014). Physical and psychosocial problems in cancer survivors beyond return to work: a systematic review. Psychooncology.

[7] Spelten ER, Sprangers MA, Verbeek JH (2002). Factors reported to influence the return to work of cancer survivors: a literature review. Psychooncology.

[8] Stergiou-Kita M, Pritlove C, Kirsh B (2016). The "Big C"—stigma, cancer, and workplace discrimination. J Cancer Surviv.

[9] Stergiou-Kita M, Grigorovich A, Tseung V, Milosevic E, Hebert D, Phan S (2014). Qualitative meta-synthesis of survivors' work experiences and the development of strategies to facilitate return to work. J Cancer Surviv.

[10] Duijts SF, Kieffer JM, van Muijen P, van der Beek AJ (2017). Sustained employability and health-related quality of life in cancer survivors up to four years after diagnosis. Acta Oncol.

[11] Hanly P, Timmons A, Walsh PM, Sharp L (2012). Breast and prostate cancer productivity costs: a comparison of the human capital approach and the friction cost approach. Value Health.

[12] Tamminga SJ, de Boer AG, Verbeek JH, Frings-Dresen MH (2010). Return-to-work interventions integrated into cancer care: a systematic review. Occup Environ Med.

[13] Zaman AG, Tytgat KM, Klinkenbijl JH, Frings-Dresen MH, de Boer AG (2016). Design of a multicentre randomized controlled trial to evaluate the effectiveness of a tailored clinical support intervention to enhance return to work for gastrointestinal cancer patients. BMC Cancer.

[14] de Boer AG, Taskila TK, Tamminga SJ, Feuerstein M, Frings-Dresen MH, Verbeek JH (2015). Interventions to enhance return-to-work for cancer patients. Cochrane Database Syst Rev.

[15] Tamminga SJ, van Hezel S, de Boer AG, Frings-Dresen MH (2016). Enhancing the return to work of cancer survivors: development and feasibility of the nurse-led eHealth intervention Cancer@Work. JMIR Res Protoc.

[16] Greidanus MA, de Boer A, de Rijk AE, Tiedtke CM, Dierckx de Casterle B, Frings-Dresen MHW (2018). Perceived employer-related barriers and facilitators for work participation of cancer survivors: a systematic review of employers' and survivors' perspectives. Psychooncology.

[17] Nilsson MI, Petersson LM, Wennman-Larsen A, Olsson M, Vaez M, Alexanderson K (2013). Adjustment and social support at work early after breast cancer surgery and its associations with sickness absence. Psychooncology.

[18] Tamminga SJ, de Boer AG, Verbeek JH, Frings-Dresen MH. Breast cancer survivors' views of factors that influence the return-to-work process—a qualitative study. Scand J Work Environ Health. 2012;38(2):144–54. 10.5271/sjweh.3199 21986836

[19] Tiedtke C, de Rijk A, Dierckx de Casterle B, Christiaens MR, Donceel P (2010). Experiences and concerns about 'returning to work' for women breast cancer survivors: a literature review. Psychooncology.

[20] Tiedtke C, Donceel P, Knops L, Desiron H, Dierckx de Casterle B, de Rijk A (2012). Supporting return-to-work in the face of legislation: stakeholders' experiences with return-to-work after breast cancer in Belgium. J Occup Rehabil.

[21] Williams-Whitt K, Bultmann U, Amick B, Munir F, Tveito TH, Anema JR (2016). Workplace interventions to prevent disability from both the scientific and practice perspectives: a comparison of scientific literature, grey literature and stakeholder observations. J Occup Rehabil.

[22] Tiedtke CM, Dierckx de Casterle B, Frings-Dresen MHW, De Boer A, Greidanus MA, Tamminga SJ, et al. Employers' experience of employees with cancer: trajectories of complex communication. J Cancer Surviv. 2017;11(5):562-77. 10.1007/s11764-017-0626-z PMC5602070 28710544

[23] Bartholomew LK, Parcel GS, Kok G, Gottlieb NH (2006). Planning health promotion programs: an intervention mapping approach.

[24] Greidanus MA, de Boer AGEM, Tiedtke CM, Frings-Dresen MHW, de Rijk AE, Tamminga SJ. Supporting employers to enhance the return to work of cancer survivors: development of a web-based intervention (MiLES intervention). J Cancer Surviv. 2020. 10.1007/s11764-019-00844-z PMC7182637 31938966

[25] Tiedtke C, Dierckx de Casterle B, de Rijk A, Christiaens MR, Donceel P (2011). Breast cancer treatment and work disability: patient perspectives. Breast.

[26] European Agency for Safety and Health at Work. Rehabilitation and return to work after cancer—instruments and practices. Luxembourg: European Union; 2018.

[27] Hees HL, Nieuwenhuijsen K, Koeter MW, Bultmann U, Schene AH (2012). Towards a new definition of return-to-work outcomes in common mental disorders from a multi-stakeholder perspective. PLoS One.

[28] Wells M, Williams B, Firnigl D, Lang H, Coyle J, Kroll T (2013). Supporting 'work-related goals' rather than 'return to work' after cancer? A systematic review and meta-synthesis of 25 qualitative studies. Psychooncology.

[29] Thabane L, Ma J, Chu R, Cheng J, Ismaila A, Rios LP (2010). A tutorial on pilot studies: the what, why and how. BMC Med Res Methodol.

[30] Blatch-Jones AJ, Pek W, Kirkpatrick E, Ashton-Key M. Role of feasibility and pilot studies in randomised controlled trials: a cross-sectional study. BMJ Open. 2018;8(9):e022233. 10.1136/bmjopen-2018-022233 PMC6169762 30257847

[31] Arain M, Campbell MJ, Cooper CL, Lancaster GA (2010). What is a pilot or feasibility study? A review of current practice and editorial policy. BMC Med Res Methodol.

[32] Eldridge SM, Chan CL, Campbell MJ, Bond CM, Hopewell S, Thabane L (2016). CONSORT 2010 statement: extension to randomised pilot and feasibility trials. BMJ.

[33] Chan AW, Tetzlaff JM, Altman DG, Laupacis A, Gotzsche PC, Krleza-Jeric K (2013). SPIRIT 2013 statement: defining standard protocol items for clinical trials. Ann Intern Med.

[34] Chan AW, Tetzlaff JM, Gotzsche PC, Altman DG, Mann H, Berlin JA (2013). SPIRIT 2013 explanation and elaboration: guidance for protocols of clinical trials. BMJ.

[35] CASTOR EDC. Available from: https://www.castoredc.com/electronic-data-capture-system/.

[36] Ministerie van Binnenlandse Zaken en Koninkrijksrelaties. Wet Verbetering Poortwachter (English: Improved Gatekeeper Act). Available from:https://wetten.overheid.nl/BWBR0013063/2008-11-01. Accessed 18 Apr 2020.

[37] Greidanus MA, Tamminga SJ, de Rijk AE, Frings-Dresen MHW, de Boer A. What employer actions are considered most important for the return to work of employees with cancer? A Delphi study among employees and employers. J Occup Rehabil. 2018;29(2):406-22. 10.1007/s10926-018-9800-z PMC6531608 30027426

[38] Hallingberg B, Turley R, Segrott J, Wight D, Craig P, Moore L (2018). Exploratory studies to decide whether and how to proceed with full-scale evaluations of public health interventions: a systematic review of guidance. Pilot Feasibility Stud.

[39] de Jong M, Tamminga SJ, de Boer AG, Frings-Dresen MH (2016). Quality of working life of cancer survivors: development of a cancer-specific questionnaire. J Cancer Surviv.

[40] de Jong M, Tamminga SJ, van Es RJJ, Frings-Dresen MHW, de Boer A (2018). The quality of working life questionnaire for cancer survivors (QWLQ-CS): factorial structure, internal consistency, construct validity and reproducibility. BMC Cancer.

[41] Tamminga SJ, de Jong M, Frings-Dresen MHW, de Boer A. The Quality of Working Life Questionnaire for cancer survivors: sufficient responsiveness for use as a patient-reported outcome measurement. Eur J Cancer Care (Engl). 2018;27(6):e12910. 10.1111/ecc.12910 30178900

[42] Bowen DJ, Kreuter M, Spring B, Cofta-Woerpel L, Linnan L, Weiner D (2009). How we design feasibility studies. Am J Prev Med.

[43] Islam T, Dahlui M, Majid HA, Nahar AM, Mohd Taib NA, Su TT (2014). Factors associated with return to work of breast cancer survivors: a systematic review. BMC Public Health.

[44] van Muijen P, Weevers NL, Snels IA, Duijts SF, Bruinvels DJ, Schellart AJ (2013). Predictors of return to work and employment in cancer survivors: a systematic review. Eur J Cancer Care (Engl).

[45] Vittinghoff E, McCulloch CE (2007). Relaxing the rule of ten events per variable in logistic and Cox regression. Am J Epidemiol.

[46] Tamminga SJ, Verbeek JHAM, Bos MMEM, Fons G, Kitzen JJEM, Plaisier PW (2013). Effectiveness of a hospital-based work support intervention for female cancer patients—a multi-centre randomised controlled trial. PLoS One.

[47] de Rijk A. Work disability prevention in the Netherlands: a key role for employers. In: MacEachen E, editor. The science and politics of work disability prevention. 1st ed: New York: Routledge; 2018. p 233-241.

[48] Kristman VL, Shaw WS, Boot CR, Delclos GL, Sullivan MJ, Ehrhart MG (2016). Researching complex and multi-level workplace factors affecting disability and prolonged sickness absence. J Occup Rehabil.

[49] Ekberg K, Pransky GS, Besen E, Fassier JB, Feuerstein M, Munir F (2016). New business structures creating organizational opportunities and challenges for work disability prevention. J Occup Rehabil.

[50] de Moor JS, Alfano CM, Kent EE, Norton WE, Coughlan D, Roberts MC (2018). Recommendations for research and practice to improve work outcomes among cancer survivors. J Natl Cancer Inst.

[51] Moore L, Hallingberg B, Wight D, Turley R, Segrott J, Craig P (2018). Exploratory studies to inform full-scale evaluations of complex public health interventions: the need for guidance. J Epidemiol Community Health.

[52] Craig P, Martin A, Browne S, Simpson SA, Wight D, Robling M (2018). Development of guidance for feasibility studies to decide whether and how to proceed to full-scale evaluation of complex public health interventions: a systematic review. Lancet.

[53] Young AE, Viikari-Juntura E, Boot CR, Chan C, de Porras DG, Linton SJ (2016). Workplace outcomes in work-disability prevention research: a review with recommendations for future research. J Occup Rehabil.

